# "Drunk or Dying" Cases

**Published:** 1903-02-21

**Authors:** 


### "DRUNK OR DYING" CASES.

THE Hon. Sydney Holland, chairman of the London Hospital, writes: The statement made in your leading article of 14th February that no attempt has been made at the London Hospital to provide beds for what are called "drunk or dying" cases—cases of emergency—is untrue. It is true that except for an "observation room," adjoining the receiving room, where drunken people are put till it is definitely settled what is the matter with them, we have no ward reserved specially for these cases. But every unoccupied surgical bed in the hospital is open for the reception of such cases, and if there is any doubt at all what is the matter, into one of these beds is the patient sent at once. So far we have never refused admission to a case which our receiving-room officer has judged proper to admit. We have also "Richmond" ward reserved for noisy cases, but not specially for these noisy ones. More than this, finding that these "drunk or dying" cases often cause inconvenience to other patients by making a noise in a general ward, we have accepted a contract for a building, I think it is 12 rooms, for "24 hours cases," as I told your editor a fortnight ago.

The gravamen of your indictment is that because special wards are not set aside for "drunk or dying" cases that thereby lives are thrown away.

My reply is that the reservation of certain wards for such cases cannot possibly lessen the risk of a dying man being turned away. At the London we have about 200,000 people every year who come to the receiving-room for help. We have six of the most skilled men in the hospital, all fully qualified, resident on duty there. But it would be miraculous if they never made a mistake, and any mistake made affords too good "copy" for any newspaper to be overlooked. You give two examples in the same issue to give force to your demand that all hospitals should provide this accommodation. But consider them, and how do they bear on the subject? In the first case two young surgeons at the Middlesex said they "were unable to say what was the matter with an injured man" and sent him to the workhouse. Assuming this to be true, they obviously committed an error in judgment and ought to have admitted the patient into the wards, and this was the verdict of the jury.

The other case was at St. Thomas's, where a woman suffering from burns had her wounds dressed by an assistant house surgeon, was made an out-patient, and attended twice as such. The coroner here said that it was admitted that the woman *would have been taken in* had her real condition been ascertained when she first attended, *but that it was not ascertained*, and the jury said that the question of admitting patients should be placed in more experienced hands. Neither of these cases have, I repeat, any bearing whatever on the question of providing an emergency ward, and I cannot understand how or why you quote them in support of your leading article. Both were instances of error of judgment, and while our men are human such errors must occur."

[Mr. Sydney Holland's letter does not meet the point, and is otherwise disappointing. He commences by describing as untrue a statement which we did not print, and then begs the question. Does Mr. Sydney Holland deny that our representative ascertained on inquiry at the hospital (see page 328 of our issue of February 7th), that no beds are specially provided for "drunk or dying" cases at the London Hospital? Does he maintain that there is no harm or risk to a certain class of cases by not having such special beds? Is he prepared to maintain that if such beds were provided the receiving-room medical officers would not admit certain doubtful cases of the type referred to, which may at present be sent away? Does he maintain that the St. Mary's case and the Middlesex Hospital case referred to by us in our articles were not doubtful cases of the "drunk or dying" type? Does he contend that it is justifiable to subject the patients in the surgical wards of the London Hospital to the disturbance and unpleasantness caused by admitting "drunk or dying" cases at any time to any vacant bed they may contain?

It is our desire to uphold and defend the administration of our voluntary hospitals. Where, however, as in the present case, defects undoubtedly exist which are dangerous to the public and damaging to the credit of the hospitals, we have a right to expect that a responsible hospital manager like Mr. Sydney Holland will frankly admit the deficiency and set about remedying it without delay. Mr. Sydney Holland's letter is disappointing too in the circumstance that it seems to shirk the responsibility for "drunk or dying" cases which ought properly to be borne by the managing committee of every hospital which does not provide emergency beds. We have been at great trouble to ascertain how far the resident medical staff are blameworthy nowadays in refusing admission to certain cases of the kind referred to, and we are satisfied that in fact the blame seldom or never lies with the receiving-room officers. These gentlemen ought therefore to be defended by the chairman of their hospital and not inferentially blamed for regrettable incidents in regard to the admission of patients, which would never arise nowadays if the resident staff had the necessary accommodation placed at their disposal.—Ed. The Hospital.]

